# Exploratory field study on the effect of Porcine circovirus 2 (PCV2) sow vaccination on serological, virological and reproductive parameters in a PCV2 subclinically infected sow herd

**DOI:** 10.1186/s12917-018-1452-x

**Published:** 2018-04-16

**Authors:** Salvador Oliver-Ferrando, Joaquim Segalés, Sergio López-Soria, Antonio Callén, Olivier Merdy, François Joisel, Marina Sibila

**Affiliations:** 1 IRTA, Centre de Recerca en Sanitat Animal (CReSA, IRTA-UAB), Campus de la Universitat Autònoma de Barcelona, 08193 Bellaterra, Spain; 2 UAB, Centre de Recerca en Sanitat Animal (CReSA, IRTA-UAB), Campus de la Universitat Autònoma de Barcelona, 08193 Bellaterra, Spain; 3 Departament de Sanitat i Anatomia Animals, Facultat de Veterinària, UAB, 08193 Bellaterra, Spain; 4 Veterinarian, Zaragoza, Spain; 5 Agronomist, Lyon, France; 6 Private veterinary consultant, Lyon, France

**Keywords:** *Porcine circovirus 2* (PCV2), PCV2 subclinical infection (PCV2-SI), Vaccine efficacy, Sow, Reproduction

## Abstract

**Background:**

This study sought to evaluate the effect of sow vaccination against *Porcine circovirus 2* (PCV2) on reproductive parameters during two consecutive reproductive cycles. The study was performed in a PCV2 subclinical infected breeding herd (PCV2 circulation but absence of major reproductive problems). Ninety-four pregnant sows were primo-immunized with a commercial PCV2 vaccine and ninety-seven were injected with phosphate-buffered saline at 6 and 3 weeks before the first studied farrowing, and then boosted at 2 weeks before the second one. Blood samples were taken throughout the study to assess PCV2 DNA load and antibodies. At farrowing, main reproductive parameters and piglet vitality index were registered. In addition, in those litters with more than three mummified or stillborn piglets, microscopic examination and PCV2 antigen detection in foetal myocardium was done.

**Results:**

Vaccinated sows showed significantly higher antibody levels compared to the non-vaccinated counterparts. PCV2 DNA was only detected at farrowing in 2 (4.2%) non-vaccinated sows. Vaccinated sows had 1.3 more live-born piglets per litter at the second cycle than non-vaccinated counterparts. Piglets from vaccinated sows had significantly higher (+ 12.7%) vitality score than the ones born from non-vaccinated sows. No PCV2 compatible lesions neither PCV2 antigen were detected in the tested foetal hearts.

**Conclusions:**

The present study represents a first attempt to demonstrate that PCV2 sow vaccination may have a positive influence on prolificacy and vitality of the offspring in a subclinical infected breeding herd. However, since reproductive outcomes at farm level may be affected by a number of factors, further studies would be needed to confirm this association.

## Background

*Porcine circovirus 2* (PCV2) is a small and ubiquitous single-stranded DNA virus with a great economic importance for the swine industry [1]. This virus is linked to a number of diseases collectively named Porcine circovirus diseases (PCVD) [2]. Among these, PCV2 has been shown to be involved in reproductive disorders in sows such as return-to-oestrus, late term abortions and increased number of mummified, stillborn and non-viable piglets at birth [3–8], as well as early embryo mortality [7, 9]. In this context, PCV2 can be transmitted to the embryos as soon as they get rid of the zona pellucida [10], to the foetus through the placenta [11] and to the new-born piglets by colostrum [12]. Under experimental conditions, some studies have demonstrated that infection of sows by both artificial insemination (AI) with PCV2-spiked semen or PCV2 inoculation can cause foetal infection and reproductive disorders [5, 13]. Moreover, foetal infections and reproductive abnormalities have been reproduced by intrauterine inoculation of foetuses or foetal liquids [14–16]. However, at farm level, it is feasible that a proportion of PCV2 effects on reproduction are linked to embryonic death [7, 10], which nowadays is still beyond the diagnostic capacity. This overall situation leads to the fact that clinical and noticeable reproductive disease (PCV2-RD) attributed to PCV2, at field level, is infrequent [6, 11, 17].

PCV2 vaccination in sows prior to farrowing is focused on the protection of the offspring by antibody transfer through the colostrum. This strategy has resulted in prevention of PCV2-systemic disease (PCV2-SD) [18], decrease of viremia [19–21] and PCV2 tissue load [22] in their progeny, and improvement of the average daily weight gain (ADWG) in subclinically infected finisher pigs [23]. Despite these facts, it has been demonstrated that sow vaccination does not fully prevent PCV2 vertical transmission [24–27].

In contrast, influence of PCV2 sow vaccination on reproductive parameters has been poorly investigated. As for example, this type of problems has been tackled as a secondary goal and in a very limited number of sows (1-3 sows per group) in some experimental studies [24, 25, 27]. At farm level, this issue has only been assessed in two peer-reviewed studies: firstly in one farm suffering from PCV2-SD in growing pigs but without evident reproductive problems (PCV2-SI scenario in the sow herd) [23] and, secondly, in one farm with PCV2-SD among weaners and serious reproductive problems in sows [28]. Whereas in the first study no significant differences on the main reproductive parameters between treatments were detected, in the second one an improvement of all measured reproductive parameters were recorded. However, information about the effect of this vaccination strategy on reproductive parameters in a farm with neither reproductive nor PCV2-SD problems (scenario resembling a situation of most breeding herds) is missing in the peer-reviewed literature. Thus, the current work was aimed to evaluate the potential effects of sow vaccination against PCV2 on serological (ELISA), virological (quantitative PCR (qPCR)) and reproductive parameters during two consecutive reproductive cycles in a PCV2 subclinical infection (PCV2-SI) scenario (PCV2 circulation in the breeding herd but absence of clinical reproductive problems).

## Methods

### Farm selection

The study was conducted in a 1900-sow farm located in Catalonia (Spain). This farm worked with weekly farrowing batches in all-in/all-out management system. The vaccination program applied by routine included sow immunization against *Porcine reproductive and respiratory syndrome virus* (PRRSV), *Aujeszky’s disease virus*, *Swine influenza virus*, *Porcine parvovirus*, *Erysipelothrix rhusiopathiae, Escherichia coli* and *Clostridium perfringens.* Piglets were vaccinated against *Mycoplasma hyopneumoniae* and PCV2 at 5 days pre-weaning and at weaning, respectively. Weaning was performed at 3 weeks of age. Moreover, no signs of any major pig diseases were present and herd immunity status against PRRSV was deemed as “positive-stable” (II-A) [29].

**Fig. 1:**
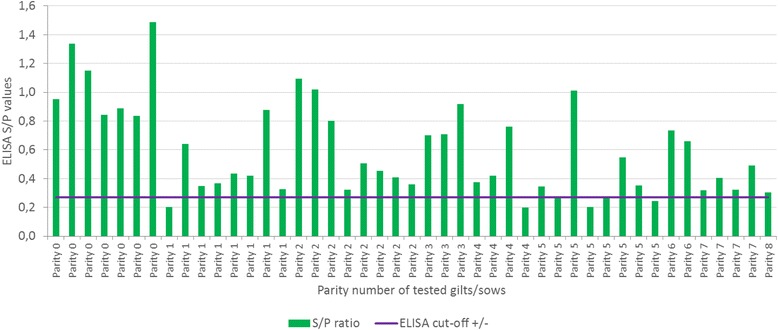
Individual PCV2 ELISA S/P results in serum samples from gilts and sows with different parity number prior to the start of the study (farm screening)

This farm had average reproductive parameter performance in line with the average Spanish national records (www.bdporc.irta.es) and did not suffer from PCVD clinical signs. Prior to the start of the study, PCV2 antibody detection was confirmed (Ingezim Circo IgG 11.PCV.K1®, Ingenasa, Madrid, Spain) in 7 out of 7 (100%) gilts and in 33 out of 38 (87%) sows of different parity that have never been vaccinated before against PCV2 (Fig. 1). In addition, antibody levels were heterogeneous (ranging from 0.20 to 1.49 ELISA S/*P* values), with a decreasing value trend in older sows.

### Study design

One hundred and ninety-one healthy sows were selected in three consecutive farrowing batches at 6 weeks pre-farrowing. These animals were individually ear-tagged and distributed in two treatment groups (Table 1). Randomization was performed according to parity (from 1 to 8) and the number of total-born and live-born piglets at the former farrowing. The comparability of the obtained groups was also checked for number of piglets weaned in the previous farrowing. Sample size was calculated using the expected differences between treatment groups in terms of magnitude of the parameters used for randomization. For this purpose, a statistical and qualitative data analysis software (GPower, University of Düsseldorf) was used. The study was conducted in two consecutive reproductive cycles. Sows were vaccinated by intramuscular injection with 2 mL of a commercial inactivated PCV2 vaccine (CIRCOVAC®, Batch number: L414725) at time points indicated in Table 1. Non-vaccinated sows received 2 ml of phosphate buffer saline (PBS) at the same time points and by the same route. Animals with different treatments were located comingled in the same gestation pens as well as in the same farrowing unit rooms.

**Table 1:** Treatment distribution of sows and vaccination schedule in both gestational cycles

Population	N^b^	Group	Treatment	Volume and doses		Number of sows bled
				First gestational cycle	Second gestational cycle	
Sows	94 (75)	V	PCV2 vaccine^a^	2 ml at 6 and 3 weeks pre-farrowing	2 ml at 2 weeks pre-farrowing	48
	97 (75)	NV	PBS			48

*V* vaccinated, *NV* non-vaccinated

^a^Animals were vaccinated with CIRCOVAC®

^b^In parentheses, number of sows remaining for the second gestational cycle in each group

Study design is represented in Table 2. Any abnormality related to general state, condition of the skin, hair and mucosa, respiratory, digestive and nervous signs, and locomotive problems was registered at different time points. At the end of the first experimental reproductive cycle, as part of routine breeder management, sows with major pathologies (lameness, injuries, etc.) and high parity (older sows) were excluded from the study. In addition, those sows showing return-to-oestrus (non-pregnant ones) in regards their counterparts, were registered and removed from the second cycle. Furthermore, sow mortality was also recorded.

**Table 2:** Study design schedule

Population	First gestational cycle				Second gestational cycle			
	6 weeks pre-farrowing	3 weeks pre-farrowing	Farrowing	Weaning	AI	2 weeks pre-farrowing	Farrowing	Weaning
Sows	CS	CS	CS	CS		CS	CS	CS
	T	T				T		
	BL		BL	BL			BL	
			RP				RP	
Piglets			S	CS			S	CS
			CS				CS	
			VI				VI	

*AI* artificial insemination, *CS* clinical signs, *T* treatment application, *BL* blood sampling, *RP* reproductive parameters assessment, *S* sex recording, *VI* vitality index

Blood samples from a randomly selected subpopulation of sows (*n* = 48 per treatment group) were taken at different time points throughout the first (at vaccination, farrowing and weaning) and second (at farrowing) gestational cycle (Table 2). Once in the laboratory, these samples were allowed clotting, and were centrifuged at 3200 rpm during 20 min at 4 °C. All sera were aliquoted and stored at − 20 °C until testing.

In each reproductive cycle, the following reproductive parameters were registered: return-to-oestrus, abortion rate, interval between expected and real farrowing date, weaning-to-fertile mating interval and number of live-born, mummified, stillborn, crushed and weaned piglets per litter.

All piglets issued from the studied sows (first and second gestational cycle) were ear-tagged, gender recorded and assigned a vitality index (VI) (see section Vitality index scoring method). Cross-fostering was only allowed within sows of the same treatment group and, when possible, within the same parity group (1st parity; 2nd-4th parity; 5th-8th parity). Moreover, from those litters with more than three mummified or stillborn foetuses, heart samples were taken and fixed in 10% neutral-buffered formalin for further histopathological analysis and PCV2 antigen detection by immunohistochemistry (IHC) (see section Histopathology and IHC in heart of foetuses). All piglets included in the study were monitored for clinical signs and mortality during lactation.

Housing conditions, feeding system, feed characteristics and health management remained consistent along the course of the trial, and were the same for both experimental groups. The present study was approved by the Ethics Committee for Animal Experimentation from the *Universitat Autònoma de Barcelona* and the Animal Experimentation Commission from the local government (*Dpt. de Medi Ambient i Habitatge from the Generalitat de Catalunya*; Reference 5796).

### Vitality index scoring method

The vitality of the piglets (within the first 3 h of life, approximately) was assessed according to a previously published index [30]. The behavioral variables evaluated were the following:

- Udder stimulation (U): 0 = no head movements, no emulating udder stimulation movements or no searching behavior within 30 s; 1 = head movements, emulating udder stimulation movements or searching behavior within 30 s.
- Number of completed circles around the enclosure (NCC): 0 = Not able to turn piglet body axis 360° from its initial orientation nor able to walk along the limits of the bucket; 1 = Able to turn piglet body axis 360° from its initial orientation or walk along the limits of the bucket once within 30 s; 2 = Able to turn piglet body axis 360° from its initial orientation or walk along the limits of the bucket at least twice within 30 s.

### Histopathology and IHC in heart of foetuses

After fixation by immersion in 10% neutral-buffered formalin and embedding in paraffin, tissue sections were processed routinely for haematoxylin/eosin (HE) staining. These samples were examined under a light microscope for the evaluation of potential microscopic lesions.

For IHC technique, serial sections (3 μm) were dewaxed in xylene and rehydrated through graded alcohols. Endogenous peroxidase was blocked with 3% of H_2_O_2_ (30%) in methanol during 30 min, followed by a 5-min wash in distilled water. Sections were then subjected to proteolytic enzyme digestion with protease type XIV (Sigma, Madrid, Spain) at a concentration of 0.1% in PBS for 8 min at 37 °C. After digestion, the sections were washed three times during 5 min in PBS. Then, these sections were incubated for 1 h at room temperature with 2% of bovine serum albumin (Sigma, Madrid, Spain) in PBS. A specific monoclonal antibody (isotype IgG2a) (Ingenasa, Madrid, Spain) against PCV2 Cap protein was applied at a 1 in 1000 dilution in 2% of bovine serum albumin in PBS (pH 7.4) to tissue sections and incubated overnight at 4 °C. Sections were then washed three times in PBS for 5 min. After washing, sections were incubated for 45 min with peroxidase-labelled polymer-horseradish peroxidase conjugated to goat anti-mouse immunoglobulins (Envision 1 System-HRP-DAB; Dako, Barcelona, Spain). Staining was completed by incubation with 3,3′diaminobenzidine chromogen solution (Envision 1 System-HRP-DAB; Dako, Barcelona, Spain). Sections were counterstained with Mayer’s haematoxylin, dehydrated, and mounted. Samples were examined under a light microscope for the analysis of immunostaining.

### DNA extraction and real-time quantitative PCR

DNA was extracted from 200 μl of serum by using the MagMAX™ Pathogen RNA/DNA Kit (Applied Biosystems) following the manufacturer’s instructions. DNA obtained was suspended in 90 μl of elution solution. To quantify PCV2 DNA in serum samples, a real-time qPCR assay (LSI VetMAX™ Porcine Circovirus Type 2-Quantification, Life Technologies) was performed. Each extraction and qPCR plate included negative controls where DNA was substituted for diethylpyrocarbonate (DEPC)-treated water. In addition, each sample reaction had an internal positive control (IPC) to monitor DNA extraction and amplification procedures. Viral concentrations were expressed as the mean log_10_ PCV2 genome copies/mL ± standard deviation (SD).

### Indirect ELISA for detecting anti-PCV2 IgG antibodies

All serum samples were tested by Ingezim Circo IgG 11.PCV.K1® assay (Ingenasa, Madrid, Spain). Optical density (OD) was measured at 450 nm by PowerWave XS reader (BioTek). Mean positive cut-off was established at 0.3 OD (± SD) following kit’s instructions (positive cut-off = OD of negative control + 0.25). ELISA results were expressed as mean S/P ratio (OD of sample/OD of positive control for each ELISA plate) ± SD.

### Statistical analyses

Statistical analyses were carried out using SAS v9.4, SAS Institute Inc., Cary, NC, USA. Significance level was set at *p* < 0.05. Proportion of animal mortality and exclusion rates between vaccinated and non-vaccinated sows was compared using Fisher exact test and Chi-square test, respectively. Reproductive parameters from all sows included in the study were analyzed and compared between experimental groups within each reproductive cycle (intra-cycle comparison) using the non-parametric Wilcoxon test. In addition, reproductive parameters from those sows farrowing in both reproductive cycles were compared between cycles (inter-cycle comparison) using generalized linear mixed models. Treatment, cycle and their interaction were considered as a fixed effects and sow as a random effect. Percentage of positive qPCR serum samples and mean log_10_ PCV2 copies/mL among those qPCR positive samples were compared at each sampling point using Fisher exact tests and Wilcoxon tests, respectively. ELISA S/*P* values were analyzed using a linear mixed model. For the analysis of VI (U and NCC), clinical signs in piglets and piglet mortality during lactation generalized linear mixed models were used. Treatment was considered as a fixed effect and sow as a random effect.

## Results

### Clinical signs in sows

No evident clinical signs were observed in any of the sows, but one, throughout the study. The particular affected sow was from the vaccinated group and developed severe lameness at the end of the first reproductive cycle, when it was excluded from the study.

### Mortality and exclusion in sows

Mortality and exclusion rates and reason of exclusion are detailed in Table 3. In both reproductive cycles, non-vaccinated sows showed numerically higher (but not significant) mortality than the vaccinated ones. Moreover, no statistically significant differences among treatment groups were observed between the two reproductive cycles with regards to the number of excluded females and the exclusion reason.

**Table 3:** Number and percentage (in brackets) of dead and excluded sows and reason of exclusion

	Treatment	V (*n* = 94)	NV (*n* = 97)
Mortality in first reproductive cycle		2 (2.13%)	5 (5.15%)
Exclusion at the end of the first reproductive cycle	Return to oestrus	13 (13.83%)	14 (14.43%)
	Culling. Old sow	2 (2.13%)	1 (1.03%)
	Culling. Lameness	1 (1.06%)	0 (0%)
	Human mistakes (vaccination, misplaced sows…)	1 (1.06%)	2 (2.06%)
	TOTAL excluded animals	17 (18.09%)	17 (17.53%)
Mortality in second reproductive cycle		0 (0%)	2 (2.67%)

No statistically significant differences (*p* < 0.05) among experimental groups were observed

*V* vaccinated, *NV* non-vaccinated

### Reproductive parameters

#### Comparison between experimental groups within each reproductive cycle (intra-cycle comparison)

Main reproductive parameters for each treatment group at first and second reproductive cycles are listed in Table 4. Number of live-born piglets at the second cycle was significantly (*p* < 0.05) higher (+ 1.3 piglets/sow) in the vaccinated group. In addition, a tendency of higher number of weaned piglets (+ 0.8) in vaccinated sows was observed (*p* < 0.1). Moreover, most reproductive parameters (return-to-oestrus [− 0.6%], interval between expected and real farrowing date [− 0.3 days] and number of mummified [− 0.1] piglets per litter) were numerically better for the vaccinated group in the second cycle. Whereas at first reproductive cycle number of stillborn piglets per litter was significantly higher in the vaccinated group, at the second period this parameter was numerically higher in the non-vaccinated one. Besides, number of crushed piglets at birth was numerically higher in the vaccinated group at first and second cycles. Regarding the abortions, there was only one abortion in each cycle for each treatment group. In addition, piglets from vaccinated sows had significantly higher vitality than the ones derived from non-vaccinated sows in both reproductive cycles. Specifically, piglets from vaccinated group showed higher percentage of head movements emulating the udder searching (statistically significant at first cycle) and greater mobility (statistically significant in both cycles) during the 30 tested seconds than the ones from non-vaccinated group.

**Table 4:** Reproductive parameters (mean ± SD) in PCV2 vaccinated (V) and non-vaccinated (NV) sows during both cycles

	First gestational cycle		Second gestational cycle	
	V (*n* = 94)	NV (*n* = 97)	V (*n* = 75)	NV (*n* = 75)
Interval between expected and real farrowing date (days)	0.45 ± 2.07^a^	0.73 ± 1.36^a^	0.72 ± 1.57^a^	1.00 ± 1.33^a^
Live-born*/litter	14.34 ± 3.24^a^	13.61 ± 3.12^a^	15.42 ± 3.43^a^	14.16 ± 3.45^b^
Crushed/litter	0.53 ± 0.84^a^	0.43 ± 0.66^a^	0.95 ± 1.16^a^	0.73 ± 1.13^a^
Mummified/litter	0.35 ± 0.62^a^	0.44 ± 0.87^a^	0.38 ± 0.70^a^	0.47 ± 0.74^a^
Stillborn/litter	1.45 ± 1.72^a^	0.88 ± 1.36^b^	0.88 ± 1.05^a^	1.24 ± 1.35^a^
Weaned/litter	12.35 ± 2.89^a^	12.06 ± 2.71^a^	12.82 ± 2.75^a^	12.01 ± 3.31^a^
Vitality index (%)**-U				
○ 1 (positive score)	91.91^a^	79.87^b^	97.14^a^	89.35^a^
Vitality index (%)**-NCC				
○ ≥ 1 (positive score)	23.40^a^	10.69^b^	22.86^a^	10.19^b^
Weaning to fertile mating interval (days)	4.51 ± 2.53^a^	4.49 ± 2.67^a^	NA	NA
Abortion (%)	0^a^	1.0^a^	1.3^a^	0^a^

Different letters in superscript mean statistically significant differences (*p* < 0.05) among experimental groups within each reproductive cycle

*NA*: not available

*including crushed piglets at birth

**This index was calculated only from piglets of three or less hours of life

#### Comparison of each experimental group between reproductive cycles (inter-cycle comparison)

In the second cycle, vaccinated sows had significantly different number of live-born (+ 1.17), crushed (+ 0.34) and stillborn (− 0.55) piglets per litter in comparison to the same sows (*n* = 75) in the first reproductive cycle. Moreover, non-vaccinated sows (n = 75) showed at their second cycle significantly higher number of crushed (+ 0.32) and stillborn (+ 0.36) piglets per litter than in their previous cycle. No statistically significant differences between cycles were observed for the rest of the parameters.

### Clinical signs and mortality in suckling piglets

In the first cycle, although the number of sows included in the study was 94 and 97 for the vaccinated and non-vaccinated groups, respectively, the number of litters monitored during the first lactation period was 93 and 96, since there was one premature farrowing and one abortion in the vaccinated and non-vaccinated groups, respectively. In this context, 9 out of 93 (9.7%) and 10 out of 96 (10.4%) litters from vaccinated and non-vaccinated sows, respectively, evidenced diarrhoea. Besides, 65 out of 1333 (4.9%) and 53 out of 1307 (4.1%) piglets from vaccinated and non-vaccinated sows, respectively, showed neurological signs clinically attributed to *Streptoccocus suis (S. suis)* infection. In the second suckling period, 8 out of 74 (10.8%) and 9 out of 75 (12%) litters from vaccinated and non-vaccinated sows, respectively, suffered from diarrhoea. In addition, prevalence of *S.suis*-like infections was reduced, recording only 3 out of 1141 (0.3%) and 2 out of 1062 (0.2%) clinical cases in piglets from vaccinated and non-vaccinated sows, respectively. No statistically significant differences between treatments in terms of diarrhoea and neurological signs were observed for any of the two lactation periods.

Piglet mortality rate (including crushed piglets around birth) in lactation was 13.9% and 16.8% for piglets from vaccinated sows at first and second cycles, respectively. Similarly, pre-weaning mortality rate in piglets from non-vaccinated sows was 11.4% and 15.2% at first and second suckling periods, respectively. However, no statistically significant differences among treatment groups were observed in any of the two lactation periods.

### Microscopic evaluation and PCV2 antigen detection in foetal heart tissues

Although, 14 litters (7.33%) from the first farrowing cycle had more than three mummified or stillborn piglets, only 2 of them were sampled. On the contrary, at second farrowing cycle, foetal heart samples from all the litters (*n* = 11, 7.33%) presenting high number of mummified or stillborn piglets were taken and evaluated. No microscopic lesions associated to PCV2 infection or PCV2 antigen were observed in myocardium of mummified or stillborn piglets from all the tested litters.

### Quantification of PCV2 DNA in sow serum samples

All vaccinated sows were qPCR negative (48 out of 48) throughout the study, whilst 2 out of 48 (4.17%) non-vaccinated sows were qPCR positive (mean viral load: 4.15 log_10_ PCV2 copies/mL) at farrowing sampling of the first reproductive cycle. No statistically significant differences between treatment groups were observed.

### Anti-PCV2 IgG antibody levels in sow serum samples

**Fig. 2:**
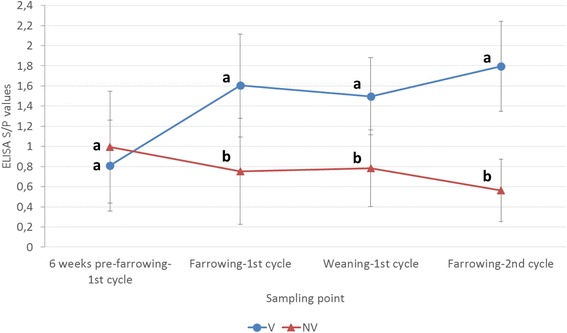
PCV2 ELISA S/P results (mean ± SD) in serum samples taken from the sows included in the study during the first (6 weeks pre-farrowing, farrowing, weaning) and second (farrowing) reproductive cycle. Different letters in superscript mean statistically significant differences (*p* < 0.05) among experimental groups at each sampling point

The course of antibodies against PCV2 for sows of the two treatment groups is shown in Fig. 2. From 6 weeks before farrowing to sampling at delivering of first gestational cycle, vaccinated group showed an increase of ELISA S/*P* values, resulting in significantly higher (*p* < 0.05) antibody levels compared to the ones from the non-vaccinated group at farrowing and weaning. In the second gestational cycle, ELISA S/P values from vaccinated sows increased again, reaching the maximum difference with the antibody levels from the non-vaccinated counterparts.

## Discussion

The objective of the present study was to evaluate the potential effect of sow PCV2 vaccination in a PCV2 subclinically infected breeding herd (PCV2 circulation but absence of overt reproductive problems). The supporting evidence of a subclinical infection was the presence of seropositive gilts and sows before starting the trial together with the low percentage of viremic animals (less than 5%) detected within the studied sow population. This low prevalence would resemble the situation of other PCV2-SI farms [31–33]. In parallel, in order to have additional information about the PCV2 infection status at the time when the study was carried out, blood samples from 12 gilts at acclimatization were taken and tested by qPCR. Indeed, PCV2 DNA was detected in 2 out of those 12 gilts (16.7%, data not shown), corroborating the PCV2-SI in the studied scenario.

Three criteria have to be fulfilled to diagnose a clinical case of PCV2-RD during late gestation [6]: 1) presence of clinical signs associated to late reproductive disorders (abortions, increased number of mummified, stillborn piglets at birth, etc.), 2) microscopic lesions in foetal heart or lymphoid tissues, and 3) detection of PCV2 antigen or DNA in those foetal tissues. In addition, return-to-oestrus problems have also been associated to PCV2-RD at early gestation [34]. The negative results of histopathology and IHC in all tested foetuses from litters with high number of mummies and stillbirths indicated that these findings were not apparently related to PCV2. Therefore, as expected, the present farm was not suffering from PCV2-RD (late reproductive failures or return-to-oestrus), since the average of all reproductive parameters were within normal ranges according to the Spanish national records.

In the present farm conditions, the PCV2 vaccination strategy applied (primo-immunization at 6 and 3 weeks pre-partum and a booster at 2 weeks before the subsequent farrowing) led to a significantly higher antibody response throughout the study period with regard to their non-vaccinated counterparts. This fact tallies with other studies where sow vaccination before mating or farrowing elicited a high antibody response [31], including neutralizing antibodies [35], in serum. In fact, the increase of antibody levels measured by the used ELISA kit is apparently correlated with PCV2 neutralizing antibody values [36], although these were not specifically measured in this study. Besides, the booster vaccination at the second cycle resulted in higher antibody levels than the ones observed after first cycle vaccination, suggesting a potential greater protection for both sows and piglets. At reproductive level, sows immunized with the PCV2 vaccine showed a significantly (*p* < 0.05) higher number of live-born piglets and tend (*p* < 0.1) to have higher number of weaned piglets per litter at the second gestational cycle. In this study, sow vaccination at first cycle was applied at a relatively late time during pregnancy when the litter size is already established; therefore, the potential impact of PCV2 vaccination on litter size was only expected after second cycle vaccination. This effect on reproductive parameters is in line with those observed by Pejsak et al. [28], but in disagreement with the ones reported by Kurmann et al. [23]. The first trial [28] was conducted in a farm with important reproductive problems (most likely related to PCV2-RD) and sporadic PCV2-SD cases. In that study, the application of a 3-year PCV2 vaccination in boars, gilts (at acclimatization) and sows (before farrowing) resulted in the improvement of all measured reproductive parameters (insemination rate, number of live-born and weaned piglets per litter and birth weight). These positive effects were more evident after several months of vaccine application, resembling the findings of the present study. On the contrary, in the other study [23], the application of PCV2 vaccine at 4 and 2 weeks before AI and 4 weeks pre-partum during 14 months in two farms with a history of recurrent PCV2-SD in growing pigs but with no apparent reproductive problems in sows did not culminate in better reproductive parameters. Therefore, to the authors’ knowledge, the current study represents the first approach in the peer-reviewed literature to show the potential benefits of PCV2 sow vaccination on reproductive parameters in a subclinically infected sow herd. It must be kept in mind, however, that reproductive performance may be influenced by other factors at farm level [37]; in consequence, although the number of studied sows is relatively high, the present study should be considered of exploratory nature and a higher number of sows and production cycles would be needed to validate obtained results.

Curiously enough, at the first farrowing post-vaccination, the number of stillborns per litter was significantly higher in the vaccinated group. The cause of this result is unknown, as there was no evidence of any factor that could adversely affect this parameter. Nevertheless, at the second gestational cycle this situation was reversed, since vaccinated and non-vaccinated groups significantly reduced and increased the number of stillborn piglets, respectively. Generally, 10 to 15% of piglets are born dead in pig farms [38]; therefore, the stillborn rate reported in the present study falls within regular values in both reproductive cycles. This parameter might be related to hypoxia during farrowing since the number of stillbirths increases in cases of high litter size, prolonged farrowing time and high birth weight [39, 40]. Besides, stillbirth can be associated with other factors such as environmental temperature, sow parity, farrowing induction, infectious diseases, mycotoxins and uterine capacity [41].

Moreover, piglets from vaccinated sows had higher vitality (in the first three hours of life) than the animals issued from non-vaccinated ones in both reproductive cycles. This finding was subjectively reported in a farm with major reproductive problems, most probably related to PCV2, using the same vaccine than the present study [28]. Besides, since VI was higher in piglets coming from vaccinated sows, one would expect to have less crushed piglets in that group. However, vaccinated sows showed higher (but non-significantly) number of crushed piglets. Most probably, this was a fortuitous event and not associated to piglet vitality, since crushing is related to other factors such as sow behaviour (depends of the sow genetics), design of farrowing crates and management practices [42].

## Conclusions

After two reproductive cycles, sows vaccinated against PCV2 experienced significantly higher antibody levels, prolificacy and vitality of their offspring. However, as reproductive performance may be influenced by multiple factors, the present study represents a further investigation of the PCV2 sow vaccination effects on reproductive parameters under a PCV2 subclinical infection scenario.

## Abbreviations

ADWG: Average daily weight gain
AI: Artificial insemination
BL: Blood sampling
CS: Clinical signs
DEPC: Diethylpyrocarbonate
DNA: Deoxyribonucleic acid
ELISA: Enzyme-linked immunosorbent assay
HE: Haematoxylin/eosin
Ig: Immunoglobulin
IHC: Immunohistochemistry
IPC: Internal positive control
NCC: Number of completed circles around the enclosure
NV: Non-vaccinated
OD: Optical density
PBS: Phosphate buffer saline
PCR: Polymerase chain reaction
PCV2: *Porcine circovirus 2*
PCV2-RD: PCV2 reproductive disease
PCV2-SD: PCV2 systemic disease
PCV2-SI: PCV2 subclinical infection
PCVD: Porcine circovirus disease
PRRSV: *Porcine reproductive and respiratory syndrome virus*
qPCR: Quantitative PCR
RP: Reproductive parameters
S: Sex recording
S. suis: *Streptoccocus suis*
S/P: Sample to positive
SD: Standard deviation
T: Treatment application
U: Udder stimulation
V: Vaccinated
VI: Vitality index
